# Development and external validation of a predictive model for in-hospital mortality in patients with liver cirrhosis and sepsis

**DOI:** 10.1038/s41598-026-43991-x

**Published:** 2026-04-02

**Authors:** Yanyu Hu, Linzhu Zhang, Jiangning Yin

**Affiliations:** 1 Nanjing Jiangning Hospital Affiliated to Nanjing Medical University, Nanjing Medical University, Nanjing, 211100 Jiangsu China; 2 Department of Oncology, Nanjing First Hospital, Nanjing Medical University, Nanjing, 210000 Jiangsu China; 3 Nanjing Jiangning Hospital Affiliated to Nanjing Medical University, No. 169, Hushan Road, Jiangning District, Nanjing, 211100 China

**Keywords:** Cirrhosis, Sepsis, Nomogram, MIMIC-IV, Bacterial infection, Liver cirrhosis

## Abstract

Cirrhosis has an increasing prevalence globally, and sepsis is a common life-threatening comorbidity of cirrhosis. The cirrhotic population benefits less from the diagnostic decision-making in current guidelines for sepsis. To establish a predictive model and validate its efficiency for predicting the risk of all-cause in-hospital mortality (IHM) in cirrhosis with sepsis. We extracted data of cirrhosis patients with sepsis from the Medical Information Mart for Intensive Care IV (MIMIC-IV) and the eICU Collaborative Research Database (eICU-CRD). The MIMIC-IV dataset was assigned at 7:3 to a training set (*n* = 1701) and an internal validation set (*n* = 729), and the eICU-CRD dataset as an external validation set (*n* = 352). Statistically, variables were screened by LASSO regression. We assessed the model performance by ROC, calibration, and decision curve analysis (DCA) curves. Finally, we compared the nomogram with the SAPS-II score and conducted DeLong tests. The model achieved AUCs of 0.783 (95% CI 0.761–0.804), 0.763 (95% CI 0.729–0.796), and 0.745 (95% CI 0.692–0.797) in the training, internal validation, and external validation sets, respectively. Calibration curves showed good agreement. Decision curve analysis demonstrated favorable clinical utility. The nomogram is valuable in early identifying high-risk groups, implementing targeted interventions, reducing IHM, and ameliorating prognosis.

**Supplementary Information:**

The online version contains supplementary material available at 10.1038/s41598-026-43991-x.

## Introduction

Cirrhosis is the 11th most frequent cause of death, with an increasing morbidity globally^1^. Sepsis, a major complication of cirrhosis, has become an important healthcare problem worldwide and is the primary cause of ICU death^2^. As noted by a clinical study incorporating 225 cohorts, cirrhosis with sepsis greatly increases short- and long-term mortality^3^. Therefore, early identifying and managing risk factors for cirrhosis with sepsis can contribute to delayed progression and higher survival rates.

The pathway analysis identified non-classical TAK1/JNK and PI3K/AKT/mTOR as important pathways in cirrhosis, and high-dimensional omics elucidated that inflammasomes^4^, inflammatory cells, and the complement system are in a state of long-term up-regulation in cirrhosis^5,6^, which greatly overlap with the known pathogenesis and progression of sepsis. In addition, sepsis and cirrhosis share similar alterations in circulatory and coagulation systems, resulting in multi-organ dysfunction^7^. Therefore, patients with cirrhosis are more prone than healthy individuals to transition from infection to sepsis.

The liver is one of the key organs damaged in sepsis. The pathophysiology of cirrhosis with sepsis involves a complex interaction of coagulation and inflammatory pathways, causing extensive vascular endothelial injury and dysregulated inflammatory response, and raising the risk of in-hospital mortality (IHM)^3,8^. Therefore, a predictive model for the short-term mortality in cirrhosis with sepsis can help improve the specificity of subsequent intervention decision-making. Several classic scoring systems can be used to assess the prognosis of patients with cirrhosis and sepsis, such as the Sepsis-related Organ Failure Assessment (SOFA), APACHE II, Model for End-Stage Liver Disease (MELD), and modified MELD-Na scores. First, the predictive value of these scores in cirrhosis with sepsis has not been specifically studied. Second, a significant number of laboratory indicators in sepsis patients with cirrhosis may deviate from the physiological ranges, resulting in non-zero baseline values in the aforementioned scores, which partially invalidates the predictive and diagnostic capabilities of these classic models. Finally, the applicability of the available scoring models in cirrhosis patients with sepsis is restricted due to their limitations in timeliness and continuity^9^. A single laboratory indicator is less accurate in predicting the outcome of cirrhosis with sepsis^10,11^. Therefore, it is necessary to establish a new model for accurately and succinctly predicting the IHM risk in cirrhosis with sepsis.

Nomograms are reliable graphical representations of predictive statistical models, which help assess disease outcomes and guide interventions^12,13^. Therefore, this study intends to create a nomogram for assessing the risk of IHM in cirrhosis with sepsis and to elucidate the possible in-hospital outcomes in cirrhosis with sepsis. The findings will serve as a supplement to the current sepsis consensus for management decision-making for the cirrhosis population, and offer guidance to clinicians for early risk assessment and subsequent interventions in the target population.

## Methods

### Data sources

In this retrospective study, we acquired data from the Medical Information Mart for Intensive Care IV (MIMIC-IV) 3.1 as the training and internal validation sets. Developed jointly by the MIT Laboratory for Computational Physiology, Beth Israel Deaconess Medical Center, and Philips, the MIMIC-IV includes a large amount of publicly accessible, multiparameter structured critical care data. We have completed the courses and training exams required to access and use the database, obtained the appropriate certificates (No. 59348300), and been granted permission to access the database. All protected health information in the MIMIC-IV^14^ had been anonymized, so no ethical review was required. Besides, the eICU Collaborative Research Database (eICU-CRD) data served as an external validation set^15^. The eICU-CRD contains information on over 20,000 hospitalizations at 208 medical centers in the U.S., and we have been granted permission to access and retrieve the database.

### Definitions

The data were acquired using pgAdmin4 by an SQL. The diagnosis of cirrhosis was reported by the International Classification of Diseases (ICD) 9/10 (5712, 5715, 5716, K703, K7030, K7031, K717, K74, K743, K744, K745, K746, K7460, K7469, P7881). Sepsis was diagnosed based on the Sepsis-3 (2016 edition)^16^, i.e., confirmed or suspected cases and a SOFA score ≥ two.

### Study population

The MIMIC-IV 3.1 and eICU-CRD were searched using SQL queries. Inclusion criteria: (1) Patients diagnosed with cirrhosis based on ICD-9 and ICD-10 codes and diagnosed with sepsis as per the Sepsis-3. Exclusion criteria: (1) Patients aged < 18 years. (2) Patients with ICU stays < 48 h. (3) Patients with HIV infections. Additionally, for multiple ICU admissions, we only extracted data of the first ICU admission. Finally, 2430 and 352 patients were enrolled from the MIMIC-IV 3.1 and eICU-CRD, respectively. The data used came from the hospitalization record, so no loss to follow-up occurred.

### Data extraction

The data were acquired using pgAdmin4, including (1) demographics: age, sex, and race; (2) baseline vital signs: temperature, heart rate, respiratory rate (RR), mean arterial pressure, and oxygenation index; and (3) comorbidities and medication: congestive heart failure, acute kidney injury, diabetes mellitus, use of glucocorticoids (GCs), use of catecholamine agents (CAs), human albumin solution, renal replacement therapy (RT), and invasive mechanical ventilation; (4) first laboratory tests post-admission: serum lactate (Lac), serum calcium, white and red blood cell counts, red blood cell distribution width (RDW), platelet count, serum total protein, serum creatinine, anion gap, aspartate aminotransferase, prothrombin time - international normalized ratio (PT-INR), alanine aminotransferase, blood glucose, serum potassium, serum sodium, and serum total bilirubin (TB); (5) disease severity: Simplified Acute Physiology Score II (SAPS-II); and (6) outcome: IHM. In-hospital survival was followed up until discharge, and the endpoints were survival at discharge and death during hospitalization. We utilized the hospitalization records of short-term hospitalized patients, so there were no censored data. All baseline data, including vital signs, laboratory parameters, and SAPS-II scores, were collected within 24 h after the first ICU admission.

### Statistical analysis

R4.4.0 was utilized for analyses. Normality of continuous variables was tested using the Shapiro-Wilk test. Variables with over 20% missing data were excluded directly, while those with less than 20% missing data underwent 5-fold multiple imputation by the “mice” package. Additionally, to validate model robustness, sensitivity analysis was conducted by directly comparing the predictive performance of the model post-imputation with that of the raw complete-case data. Random forest (method = “rf”) was utilized for the specific imputation algorithm, with a fixed random seed (seed = 123) to ensure reproducibility of the imputation process. To mitigate the impact of extreme values on the model, Winsorization was applied to handle outliers, i.e., values below and above the corresponding threshold values were replaced with the 1st percentile and 90th percentile. Prior to modeling, the MIMIC-IV dataset was randomized at 7:3 into a training set (*n* = 1701) and an internal validation set (*n* = 729) using the R sample function. To ensure reproducibility, a fixed random seed (seed = 2024) was set during randomization. Variables were selected in the training set using LASSO regression (the R “glmnet” package). The optimal penalty parameter (λ1se) was determined by 10-fold cross-validation. To balance predictive performance and simplicity of the model, the one-standard-error criterion (λ1se) was ultimately selected for predictor screening. This effectively prevented overfitting using moderate compression coefficient, creating a streamlined model with stronger clinical utility. Based on the predictors screened, a nomogram predicting mortality risk in cirrhosis with sepsis was created by the “rms” package. Model performance was evaluated in the training set and internal/external validation sets: The discrimination capability was assessed by calculating the area under the receiver operating characteristic (ROC) curve (AUC) using the “pROC” package. The agreement between predicted and actual probabilities was assessed by calibration curves. Finally, decision curve analysis (DCA) was performed using the “rmda” package to assess the model’s net benefit and utility in clinical use. Additionally, to further validate the clinical value of the nomogram model, it was compared with the traditional Simplified Acute Physiology Score II (SAPS-II). The AUC was compared between the nomogram model and SAPS-II across datasets using the DeLong test, thereby assessing whether the improvement in predictive performance was statistically significant. In the external validation set, a subgroup (*n* = 114) with both SAPS-II and APACHE-II scores was identified to complete DeLong tests for nomogram, SAPS-II, and APACHE-II.

### Construction and validation of nomogram

Finally, 2430 patients (MIMIC-IV) were assigned at 3:7 to an internal validation set (*n* = 729) and a training set (*n* = 1701), while 352 patients (eICU-CRD) as an external validation set. Predictor variables for cirrhosis with sepsis were screened by LASSO regression. The model was refined by establishing a penalty function to make the model more concise and retain the advantage of subset shrinkage. The validation of the model consisted of the following four items: (1) AUC for assessing the model’s discriminatory power; (2) calibration curves for assessing the model’s calibration property; (3) DCA curves for assessing the clinical utility of the nomogram by the net benefit at different threshold probabilities; and (4) comparison of nomogram and the SAPS-II score.

## Results

### Baseline characteristics and sensitivity analyses pre- and post-data imputation

**Fig. 1:**
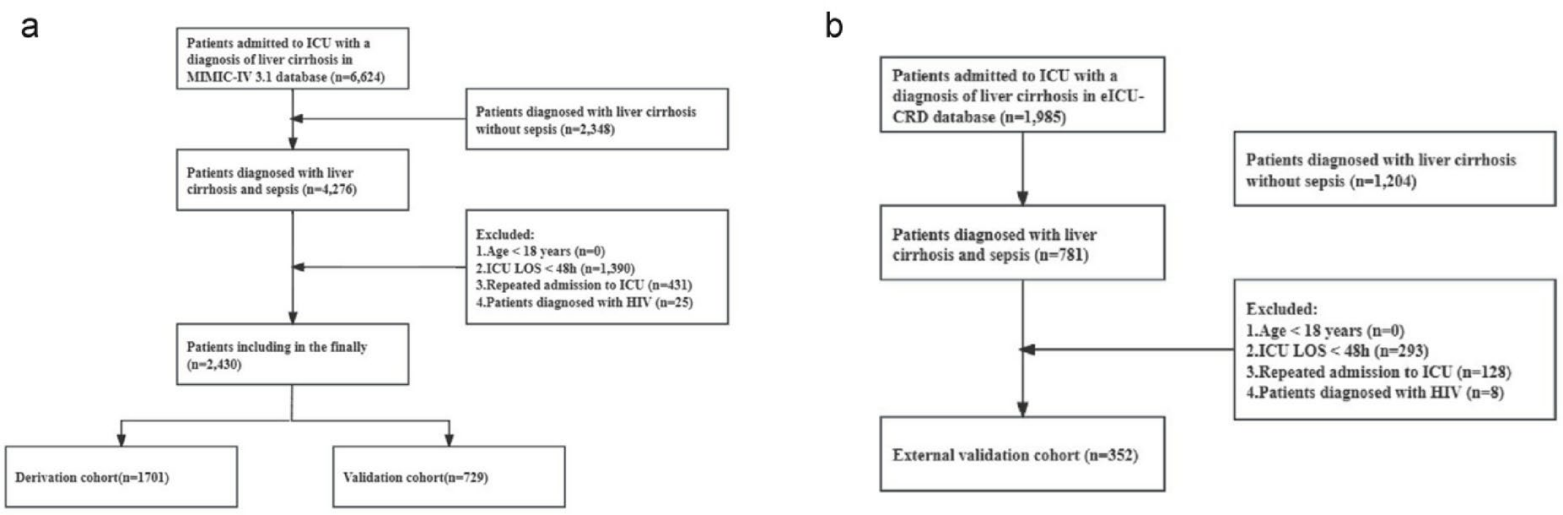
Flowchart of screening.

We finally set up a training set (*n* = 1701), an internal validation set (*n* = 729), and an external validation set (*n* = 352) (Fig. 1a, b). Table 1 presents baseline demographic characteristics of the training set, internal validation set, and external validation set (eICU-CRD). As shown by the P-values, the indicators had statistically significant differences among the three sets, except for WBC, RDW, RBC, creatinine, INR, potassium, sodium, and SAPS-II, suggesting significant heterogeneity in the populations from the two independent databases, MIMIC-IV 3.1 and eICU-CRD. To validate the influence of data imputation, sensitivity analyses were additionally conducted pre- and post-imputation. The results revealed highly consistent model performance across different data processing approaches. The AUC of the training set post-imputation was 0.783 (95% CI 0.762–0.804), roughly consistent with that of the raw complete-case data (0.777, 95% CI 0.756–0.797) (Supplementary Table S2 and Fig. S1). This suggests that variable selection and predictive performance were almost impervious to the imputation process.

**Table 1:** Baseline characteristics of patients from the MIMIC-IV 3.1 and eICU-CRD.

	[ALL]	Derivation	External validation	Internal validation	*P*.overall
	*N *= 2782	*N *= 1701	*N *= 352	*N *= 729	
Sex	0.36 (0.48)	0.36 (0.48)	0.45 (0.50)	0.33 (0.47)	0.001
Age	58.9 (12.1)	59.4 (12.2)	56.7 (10.9)	58.8 (12.3)	<0.001
Race	0.33 (0.47)	0.35 (0.48)	0.22 (0.41)	0.35 (0.48)	<0.001
AKI	0.87 (0.34)	0.94 (0.24)	0.42 (0.49)	0.91 (0.28)	<0.001
CHF	0.19 (0.39)	0.21 (0.41)	0.02 (0.14)	0.21 (0.41)	<0.001
DM	0.28 (0.45)	0.31 (0.46)	0.07 (0.26)	0.29 (0.45)	<0.001
Mechanical.ventilation	0.70 (0.46)	0.73 (0.44)	0.48 (0.50)	0.72 (0.45)	<0.001
RT	0.25 (0.43)	0.30 (0.46)	0.01 (0.12)	0.27 (0.44)	<0.001
Use.of.GCs	0.45 (0.50)	0.48 (0.50)	0.23 (0.42)	0.47 (0.50)	<0.001
Use.of.CA	0.72 (0.45)	0.77 (0.42)	0.38 (0.49)	0.75 (0.44)	<0.001
Use.of.HAS	0.73 (0.45)	0.75 (0.43)	0.47 (0.50)	0.79 (0.41)	<0.001
Temperature	36.6 (0.97)	36.7 (0.90)	36.6 (1.10)	36.6 (1.05)	0.005
RR	20.0 (5.38)	20.0 (5.04)	20.9 (7.57)	19.5 (4.79)	0.004
MBP	77.7 (19.1)	78.3 (19.0)	72.7 (17.8)	78.6 (19.7)	<0.001
Lac	3.54 (3.45)	3.41 (3.23)	4.51 (4.25)	3.40 (3.45)	<0.001
WBC	12.0 (8.53)	12.0 (8.26)	12.5 (9.75)	12.0 (8.54)	0.597
RDW	17.6 (3.08)	17.6 (3.00)	17.6 (3.50)	17.6 (3.06)	1
Platelets	122 (90.2)	123 (85.0)	107 (57.8)	128 (112)	<0.001
RBC	3.02 (0.76)	3.00 (0.75)	3.05 (0.77)	3.06 (0.79)	0.165
Albumin	3.25 (1.16)	2.91 (0.70)	5.57 (1.05)	2.94 (0.70)	<0.001
Creatinine	1.98 (1.70)	2.00 (1.69)	2.06 (1.77)	1.90 (1.67)	0.283
Anion.Gap	16.2 (5.97)	16.6 (5.85)	13.5 (6.28)	16.4 (5.78)	<0.001
INR	2.07 (1.04)	2.08 (1.01)	1.98 (0.99)	2.09 (1.11)	0.192
ALT	91.0 (121)	95.5 (128)	65.2 (68.7)	93.1 (125)	<0.001
AST	355 (1210)	399 (1233)	135 (141)	359 (1415)	<0.001
Total.Bilirubin	7.05 (9.01)	7.23 (9.34)	5.21 (4.98)	7.55 (9.61)	<0.001
Potassium	4.27 (0.88)	4.29 (0.88)	4.18 (0.86)	4.27 (0.89)	0.079
Sodium	136 (6.50)	136 (6.71)	136 (6.36)	135 (6.03)	0.034
SAPS.II	68.8 (25.8)	68.4 (24.3)	72.5 (32.3)	68.1 (25.7)	0.06
STATUS	0.45 (0.50)	0.42 (0.49)	0.61 (0.49)	0.44 (0.50)	<0.001

Note: STATUS refers to whether the patient died in the hospital. AKI, acute kidney injury; CHF, congestive heart failure; DM, diabetes mellitus; Mechanical ventilation, mechanical ventilation; HAS, human albumin injection; Use of GCs, use of glucocorticoids; Use of CA, use of catecholamines; SAPS-II, simplified acute physiology score.

### Nomogram

**Fig. 2:**
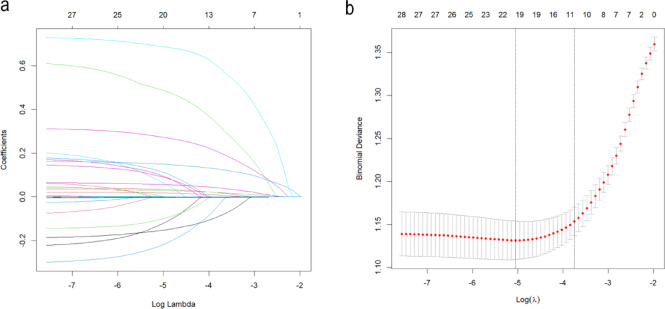
Lasso regression path and cross-validation for predictor variable selection. **a** The path of Lasso coefficients for the 28 predictors is displayed. Each curve traces a coefficient across the logarithm of the lambda (λ) regularization parameter. From left to right, coefficients converge towards zero as λ rises. This demonstrates how Lasso regularization influences predictors. The prediction error is minimized by the optimal λ value by striking a bias-variance balance. **b** Cross-validation for optimal λ. The y-axis indicates the mean square error (MSE) against the logarithm of λ on the x-axis. Red points indicate the MSE for specific λ, with the error bars representing its standard error. Vertical dashed lines highlight the λ achieving the minimum MSE (left) and the λ selected by the one-standard-error rule (right). Following cross-validation, the optimal λ is chosen to maximize predictive performance while keeping the model simple.

**Fig. 3:**
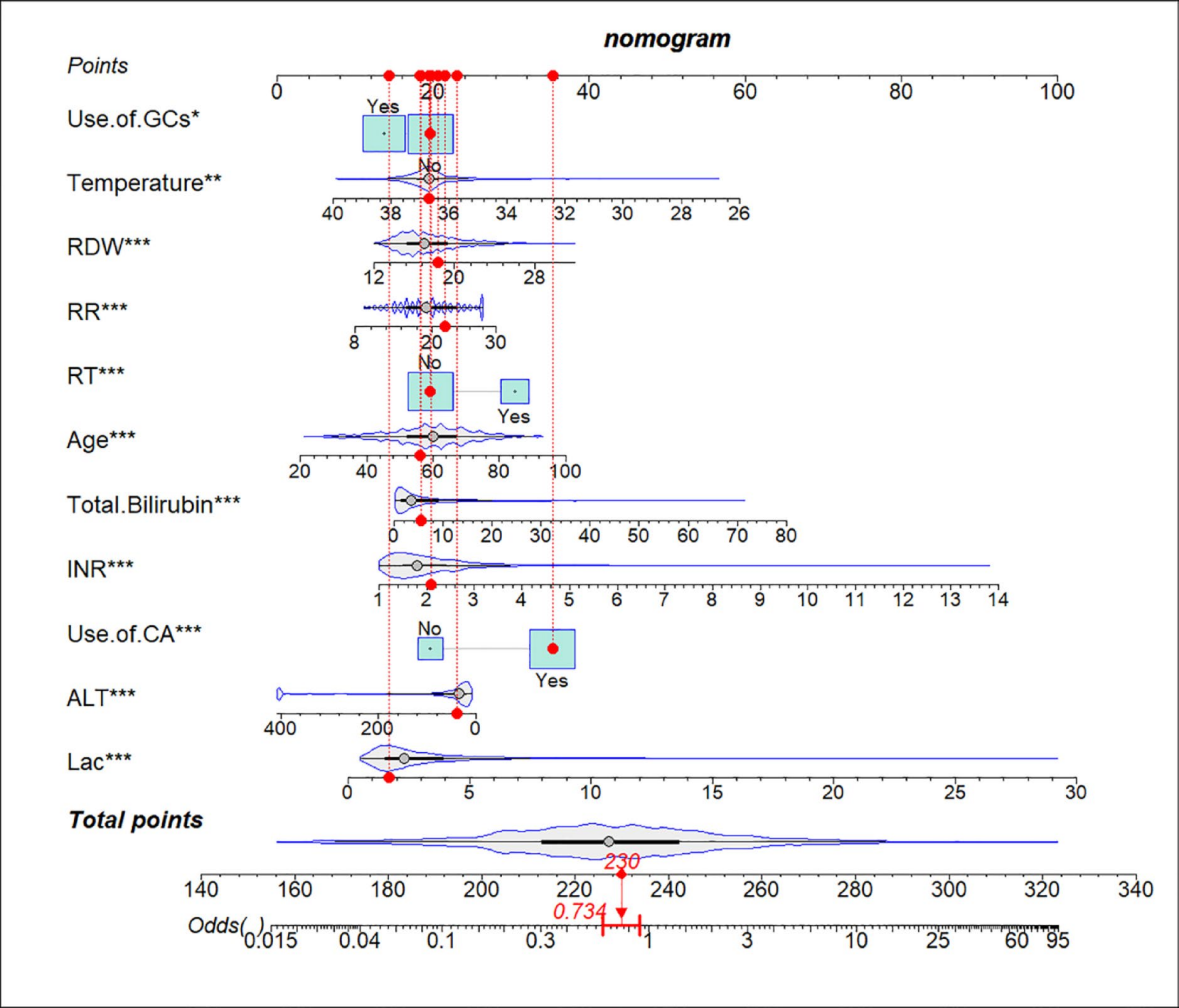
Nomogram for predicting the risk of IHM in cirrhosis with sepsis. The score for each variable corresponded to the point on the axis, and the total score on the lower axis corresponded to the risk of IHM.

The initial variables in the training set were optimized by LASSO regression, and 11 potential predictors (age, ALT, INR, Use. of. GCs, Use. of. CAs, Temperature, Total. Bilirubin, RR, RT, RDW, and Lac) for the prognosis in cirrhosis with sepsis were ultimately identified as the optimal set (Fig. 2a, b). The changes in coefficients of variables were compared before and after LASSO regression (Table S1), and the variables were selected when λ = 1se. We enhanced transparency in variable screening by Table S5 Multivariable logistic regression analysis of predictors selected by LASSO. To visualize the model, we developed a nomogram model (Fig. 3), with the score for each variable corresponding to the point on the axis, and the total score on the lower axis corresponding to the risk of IHM.

### Validation and assessment of nomogram

#### Internal validation in MIMIC-IV Cohort

**Fig. 4:**
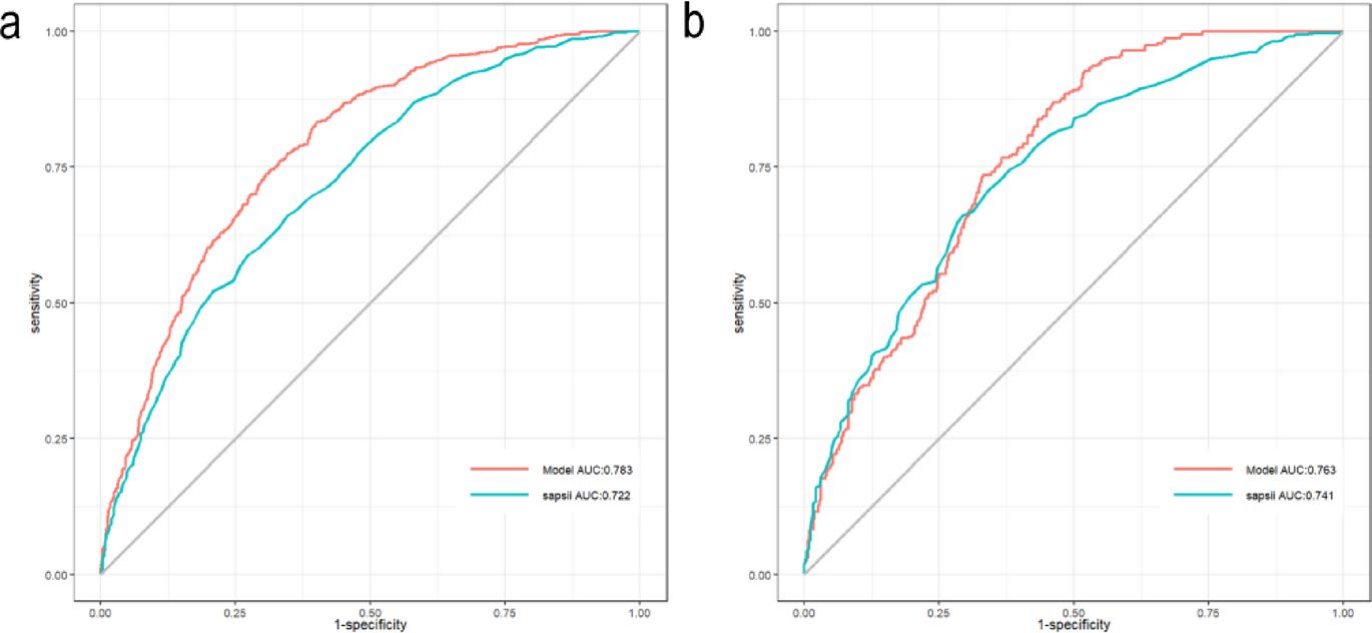
ROC curves for nomogram and SAPS-II. **a** Training set; **b** internal validation set.

**Fig. 5:**
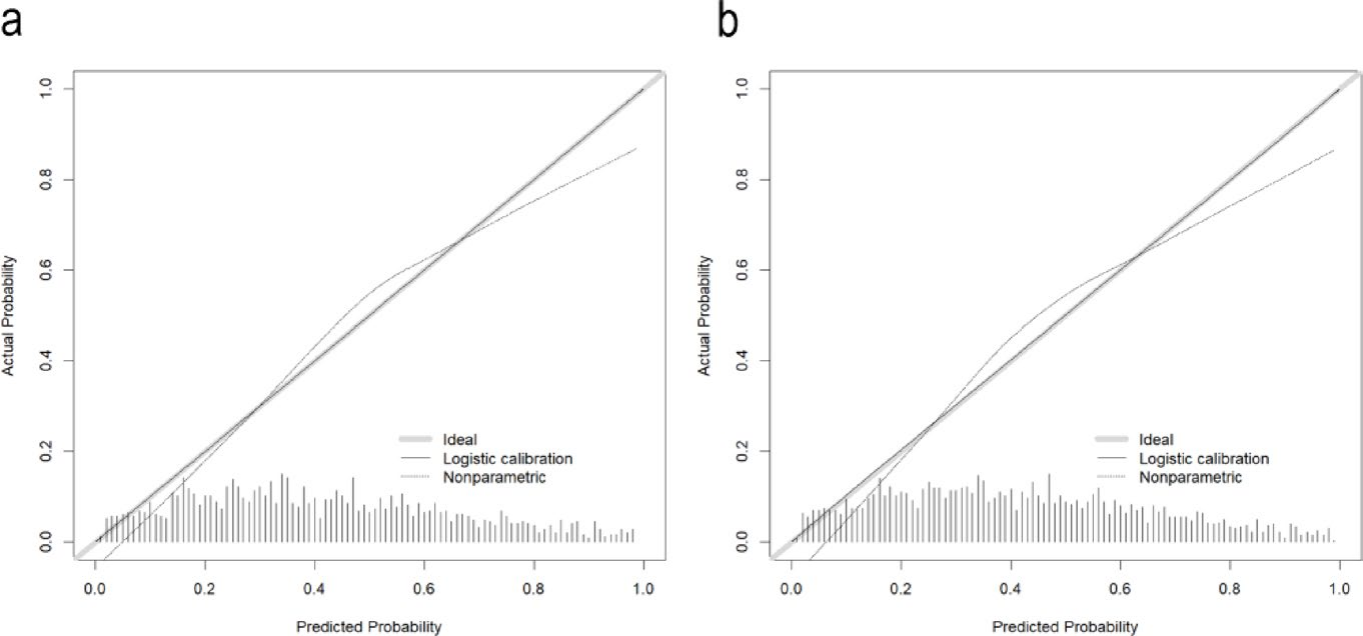
Calibration curves for nomogram and SAPS-II. **a** Training set; **b** internal validation set.

**Fig. 6:**
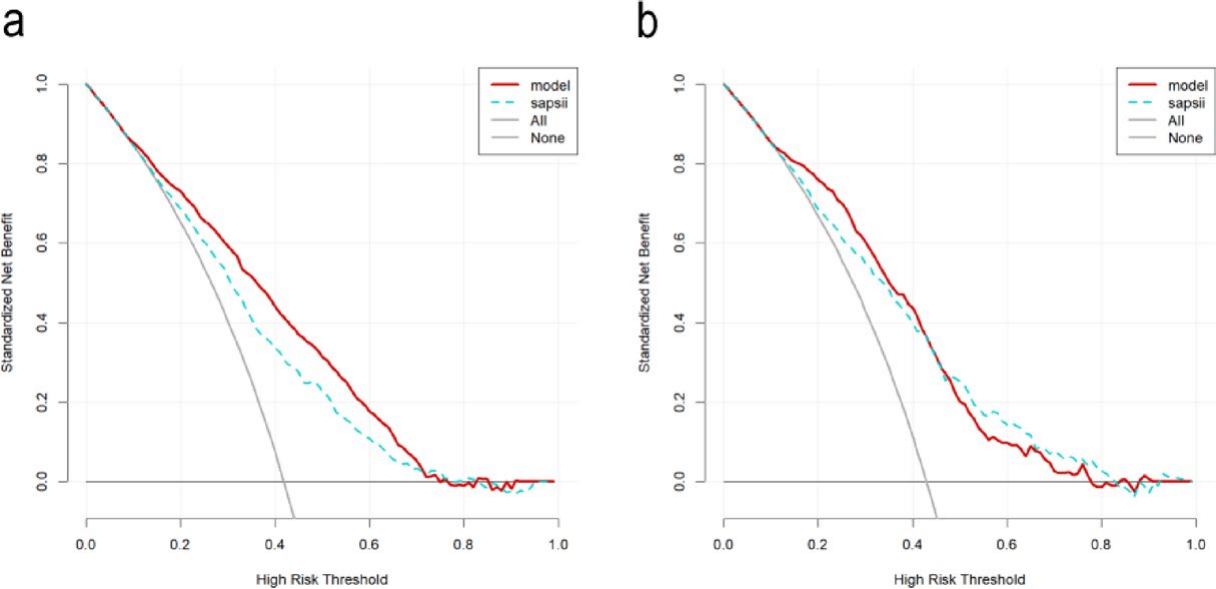
Decision-making curves for nomogram and SAPS-II. **a** Training set; **b** internal validation set.

We conducted the internal validation of the nomogram. The nomogram’s predictive performance was assessed by ROC curves, and the AUCs were 0.783 (95% CI 0.761–0.804) and 0.763 (0.729–0.796), respectively, for the training and internal validation sets, far higher than the SAPS-II (Fig. 4a, b). The results demonstrate the model’s good discriminatory power for prognosis. The calibration curve revealed high agreement between predicted and actual results in the two sets (Fig. 5a, b). As displayed by the DCA curve, the nomogram predicted the clinical benefit in the two sets. Finally, we compared the nomogram with the SAPS-II score (Fig. 6a, b). To sum up, the nomogram model demonstrated comparable discriminatory power to the SAPS-II score while offering a more streamlined and practical approach for clinical risk assessment.

#### External validation in eICU-CRD cohort

**Fig. 7:**
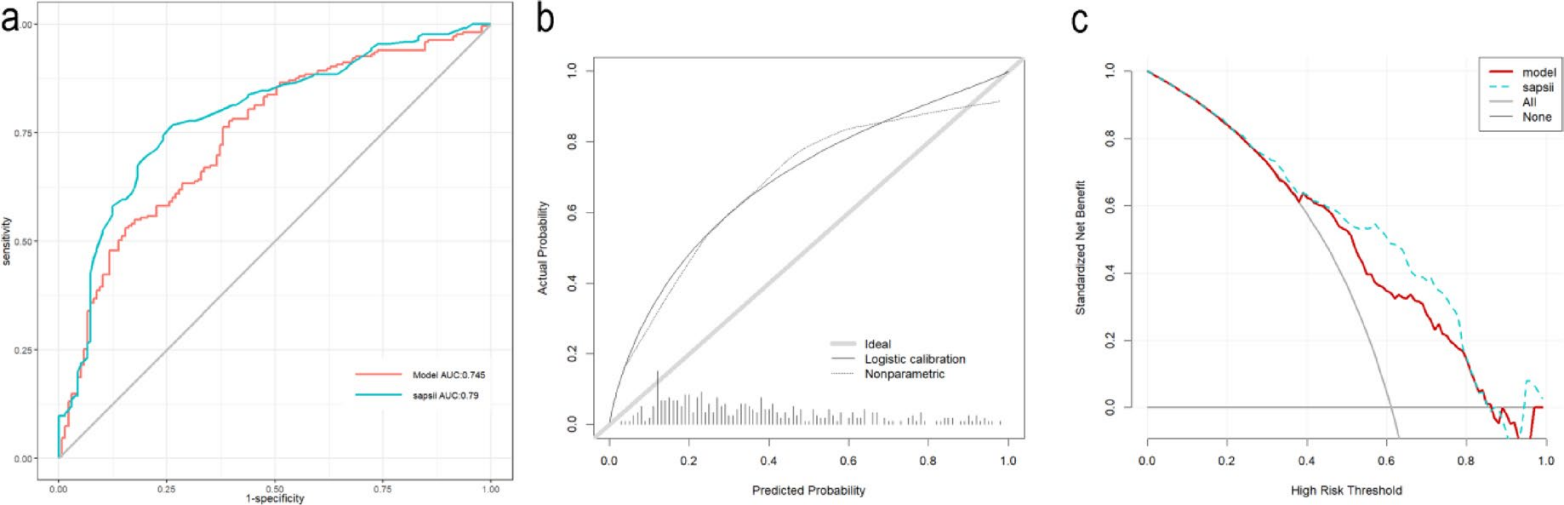
Validation of external set. **a** ROC curve; **b** calibration curve; **c** decision-making curve.

We conducted the external validation of the nomogram. The predictive performance of the nomogram was assessed by ROC curves, and the AUCs was 0.745 (0.692–0.797) in the external validation (Fig. 7a). The calibration curve revealed an acceptable agreement between predicted and actual results in this set (Fig. 7b). As displayed by the DCA curve, the nomogram predicted the clinical benefit in this set. Finally, we compared the nomogram with the SAPS-II score (Fig. 7c). In the eICU-CRD external validation cohort, the DCA curve indicated that the performance of our nomogram was not inferior to the SAPS-II score.

#### Statistical comparison of the nomogram with existing scoring systems

The discriminatory power was compared between nomogram and SAPS-II by the DeLong test. In the training set, the nomogram had a significantly higher AUC value than SAPS-II (0.783 vs. 0.723, *P* < 0.001). In the internal validation set, the nomogram’s AUC value remained high, but the difference was not statistically significant between the two (0.763 vs. 0.741, *P* = 0.287) (Supplementary Table S3). This suggests that this model had comparable predictive performance to the gold standard score but did not outperform the SAPS-II score. Furthermore, to assess model robustness, the nomogram, SAPS-II, and APACHE-II were compared for the performance in a subgroup (*n* = 114, with complete scoring indicators) of the external validation set. The nomogram’s AUC (0.798) was superior to both SAPS-II (0.737) and APACHE-II (0.728), with no statistically significant differences due to the limited sample size in the subgroup (*P* = 0.196 and 0.138). However, the superiority in consistency demonstrated the model’s great potential as a simplified early warning tool (Supplementary Table S4).

#### Sensitivity analysis excluding intervention variables

To eliminate potential circular reasoning bias from intervention variables (e.g., Use. of. CAs, RT, Use. of. GCs) during hospitalization, a sensitivity model incorporating only baseline data immediately post-admission was constructed (Fig. S2). The results revealed that after excluding all intervention variables, the model maintained robust predictive performance in the internal validation set (AUC 0.785) (Supplementary Figures S3-S6). Additionally, the DCA showed that the model still provided significant net benefit for clinical decision-making without relying on treatment feedback information. This suggests that the core biological indicators of this model possessed independent and robust predictive value early post-admission.

## Discussion

Based on the large-sample data analysis, a nomogram is a reliable, concise, and practical predictive tool by intuitively presenting the contribution rate of each factor. Nomogram has been widely applied to the circulatory system^17^, surgery, and oncology^18^, obtaining good clinical effects. The MIMIC-IV^14^ and eICU-CRD^15^ are both authoritative databases in critical care medicine, with massive data applied to medical-surgical emergency research^17–19^. Using the above statistical methods and dataset sources, Lin et al.^20^ carried out pioneering work in this field. Due to the homogeneity of a single-center data source, however, the robustness of their model across diverse healthcare settings remains to be further investigated. On this basis, this study trained the model using the latest MIMIC-IV3.1 dataset. By integrating the eICU-CRD, a large-scale, nearly nationwide, multi-center database, we conducted rigorous external validation and sensitivity analyses. This not only verified the model’s robustness across regions and centers but also provided more universally applicable evidence for clinical practice. Through comprehensive analysis of data on patient demographics, comorbidities, ICU invasive treatment, and laboratory tests, we identified 11 predictors that were significantly associated with the outcome: use of glucocorticoids, use of catecholamine agents, renal replacement therapy, age, temperature, respiratory rate, red blood cell distribution width, TB, serum lactate, prothrombin time - international normalized ratio, and alanine aminotransferase.

This study intends to develop a prognostic prediction model specifically for cirrhosis patients with sepsis. The model incorporated 33 clinically accessible and cost-effective indicators, and its robustness was verified by internal and external validation in two independent electronic databases with significant heterogeneity.

When establishing the model, we specifically incorporated multiple indicators fully reflecting hepatic function, including indicators of hepatic synthetic capacity (albumin, INR), metabolic capacity (TB), and sensitivity to hepatocyte injury (ALT, AST). Compared with general scoring models like SOFA, which lack liver-specific indicators, this model possessed higher specificity and predictive efficacy when assessing sepsis patients with cirrhosis. Clinically, the indicators of this model can be easily and rapidly collected at the bedside to facilitate real-time dynamic monitoring and assessment by frontline clinicians, effectively making up for the limitation of APACHE-II and GCS in timeliness. Notably, patients across the entire spectrum of cirrhosis were successfully included in this study, enabling this model to apply to different stages of liver disease and overcoming the deficiency of MELD or MELD-Na which is only restricted to end-stage liver disease. The clinical significance of the selected indicators will be discussed in detail in the following paragraphs.

CAs, represented by norepinephrine, are routinely used as first-line treatment for restoring hemodynamics in the management of shock patients. However, many recent studies^21^ suggest that patients with sepsis do not generally benefit from CAs. In patients with sepsis, blindly using CAs may produce an “off-target” effect, raise the risk of cardio-cerebrovascular events, interrupt intercellular oxygen delivery, and accelerate multi-organ failure. Norepinephrine, the first-line CA for correcting blood pressure, possesses an overall “immunosuppressive” effect. Its ability to weaken the generation of ROS and inflammatory mediators in mouse models and patients has been verified^22^, which interferes with immune responses in both mice and humans, and further enhances sepsis-induced immune paralysis. Meanwhile, epinephrine and norepinephrine bind to cells in patients with cirrhosis^23^ to trigger a cellular response and production of excess extracellular matrix by hepatic stellate cells^24^, worsening cirrhosis. More interestingly, in addition to immune paralysis, norepinephrine directly contributes to the growth of gram + and gram- bacteria in vitro^25^. The above mechanisms demonstrate that use of CAs is positively associated with increased sepsis mortality. Against the background of aberrantly enhanced basal sympathetic nerve activity and aberrantly high sensitivity of catecholamine receptors in patients with cirrhosis, exogenous catecholamines are often administered to correct sepsis-induced shock. Patients are highly susceptible to malignant cardiovascular events, immune paralysis, and multi-organ failure in the early stage. In addition, CAs are applied to patients with circulatory system dysregulation and a high risk of IHM. To sum up, use of CAs is positively associated with the risk of IHM in cirrhosis with sepsis.

Renal failure is the most frequent complication of cirrhosis and is closely related to the prognosis. AKI in cirrhosis is attributed to various etiologic factors, and hepatorenal syndrome produces the worst outcome. Cirrhosis with sepsis is at high risk of AKI, and it can be treated with RT to reduce circulatory load and eliminate inflammatory mediators, producing therapeutic benefits. Unfortunately, several reports^26^ documented that RT fails to greatly improve the in- and out-of-hospital survival status. In this study, RT-treated patients were in a critical condition and had insufficient hepatic and renal compensatory capacity, suggesting a poor prognosis. Therefore, RT was positively associated with the IHM, consistent with previous reports and clinical practice.

High age usually acts as an independent risk factor for critical illness. A review of clinical data of sepsis and transcriptomic analyses^27^ revealed that immune senescence has a negative correlation with the activation of vascular endothelial cells, and far fewer genes are involved in leukocyte activation and adaptive immunity in the elderly than in the population aged below 50 years; immune senescence has a positive correlation with the degree of endothelial injury downstream of the inflammatory response, and as can be seen from the quantification of classical cytokine concentrations, the incidence of injurious inflammatory events is higher in the elderly than in the population aged below 50 years. Our nomogram model confirmed the positive association of age with the increased risk of IHM. We believe that cirrhosis with sepsis in elderly patients is characterized by low immune response and high incidence of downstream endothelial injury, as well as delayed onset of clinical symptoms, rapid progression of multi-organ failure, and unfavorable prognosis. To sum up, clinicians should assess and intervene in elderly patients as early as possible to reduce the risk of IHM. In addition, coagulation dysfunction often interacts with the inflammatory response in sepsis, and cirrhosis patients are in a long-term state of chronic coagulation activation and secondary excessive fibrinolysis^28^. When cirrhosis with sepsis occurs, complex inflammatory and coagulation responses interact in vivo^29^, manifested by a prolonged PT-INR corresponding to a poor prognosis. Our model aligned with the pathophysiologic progression of cirrhosis with sepsis.

Lac is a reliable marker for organ failure and death in sepsis^30^. Microcirculatory disturbances are a key contributor to elevated Lac levels. The liver undertakes 60% of Lac metabolism and is highly susceptible to sepsis-induced circulatory disturbances, consistent with the findings of this study. Moreover, Lac accumulation worsens metabolic acidosis and leads to hyperpnea, manifested by a significant elevation of RR, suggesting an increased risk of death. Similarly, we found by the nomogram that RR was linked to the IHM. In addition, RDW is a rapid, simple, and easy-to-access biomarker that can guide clinicians in early risk stratification. An RCT^31^ that included 302 subjects revealed that RDW and its derivatives are sensitive markers for the diagnosis of alcoholic cirrhosis, with cutoffs of 0.912 (> 14.2%), 0.965 (> 0.075), and 0.914 (> 8.684) for RDW, RPR, and RLR, respectively. RDW also performs well in predicting the mortality risk in sepsis^32^. Therefore, the mortality risk in cirrhosis with sepsis is associated with increased RDW, consistent with the predictive result of our model. This model identified low temperature as a significant predictor of mortality, consistent with previous cohort studies^33^.

Specifically, an experimental study^34^ confirmed that cirrhosis patients with sepsis have significantly lower mean blood temperature than non-cirrhosis patients (35.5 ± 0.6 °C vs. 37.6 ± 0.2 °C, *P* < 0.05). The clinical significance of this variation lies in the fact that body temperature is indirectly indicative of normal or abnormal tissue perfusion and also serves as a common assessment tool for early inflammatory responses. However, in cirrhosis patients, prolonged deposition of immune complexes in dysfunctional hepatocytes leads to depletion of inflammatory mediators, so the body is predisposed to “immune paralysis”. When experiencing rapidly progressing, multi-organ diseases like sepsis, activation of the immune network becomes time-dependent, and classic inflammatory pathways face the risk of inflammatory mediator exhaustion during downstream protein events. Clinically, this is manifested as bypassing the typical phase of temperature elevation and rapidly progressing to a critical condition characterized by circulatory failure and hypoperfusion^35^. Therefore, low temperature is not a benign indicator in cirrhosis, but rather signals a high-risk condition and elevated IHM.

Both ALT and TB reflect hepatic function, with their elevation often indicating hepatocyte damage and impaired function. In the nomogram, elevated TB predicted high mortality risk, a conclusion supported by prior studies^36^. First, cirrhosis patients inherently have a significantly lower proportion of functional hepatocytes than non-cirrhosis patients, and their diminished capacity to eliminate bilirubin (a metabolic product) results in bilirubin accumulation. Meanwhile, relevant research^37^ suggests that hyperbilirubinemia may worsen systemic inflammation by oxidative stress mechanisms, thereby promoting the dysregulation of the inflammation-coagulation interaction in cirrhosis patients. These cascade positive feedbacks often indicate high mortality rates, theoretically supporting our experimental conclusions.

Notably, low ALT levels predicted a high mortality risk in the nomogram, contrary to conventional disease progression. The results of reviewing meta-analyses and RCTs showed that the proportion of patients with normal ALT levels cannot be ignored in those with liver injury^38^. Verma et al. found that the AUROC values are 0.62 and 0.46 for ALT levels associated with cirrhosis, suggesting that ALT is not the optimal predictor for NASH or late fibrosis^39^. Therefore, previous studies have revealed that ALT levels exhibit limited explanatory power regarding hepatic function in cirrhosis patients. For the characteristics of cirrhosis, few functional hepatocytes remain in cirrhosis patients, and a large number of hepatocytes have been replaced with fibrous tissues, leading to severe depletion of hepatic functional reserve. In this context, ALT release induced by hepatocyte necrosis is less significant even in sepsis, and ALT levels may be normal or even low due to a decrease in total hepatocyte count. In such cases, therefore, low ALT is not indicative of normal liver function, but rather signals end-stage liver failure with extremely poor prognosis.

The above 11 predictors were determined to be the risk factors for the IHM in cirrhosis with sepsis. The predictive model exhibited good discriminatory power, calibration property, and clinical decision-making capability in both internal and external validation. Since we utilized the MIMIC-IV 3.1 and eICU-CRD, our findings are generalizable.

Multiple imputation was utilized to handle missing data, preserving the maximum sample size and reducing bias. Therefore, sensitivity analysis was conducted by comparing the performance of the model post-imputation and the raw complete-case data. The results confirmed highly consistent discriminatory power (AUC) between the two (Table S2; Fig. S1). This suggests that multiple imputation did not distort the intrinsic relationships among variables, and it also enhanced the model’s robustness in clinical practice, as missing data are inevitable in real-world clinical settings.

In comparative analysis, the DeLong test results revealed that the model performance in the validation set was comparable to SAPS-II (*P* = 0.287), rather than statistically superior to SAPS-II. This underscores the reliability of SAPS-II as a classic scoring system in intensive care medicine. Although the AUC value of the nomogram improved compared with SAPS-II and APACHE-II in the external validation subgroup, this model was positioned as a practical alternative to early screening rather than a replacement for comprehensive assessment systems such as SAPS-II. We also acknowledge that the interpretability of small-sample data is limited and requires further validation in future multicenter studies.

To eliminate potential circular reasoning bias from intervention variables (e.g., Use. of. CAs, RT), we conducted the sensitivity analysis. The results showed that the baseline model excluding all intervention variables exhibited robust predictive capability (Fig. S3–S6), with the trends of primary variables highly similar to the original model. This confirms the model’s core predictive value originates from patients’ physiological basis at admission.

In this study, intervention variables were preserved in the master model based on the consideration of clinical practice. The condition of sepsis with cirrhosis is highly dynamic, and patients’ response to CAs or GCs within 24 h after ICU admission can accurately capture the direction of disease progression. Thus, the nomogram designed in this study possessed dual clinical value: It enabled immediate early warning using baseline indicators at admission, and also achieved dynamic correction by feedback post-intervention. This design provides ICU clinicians with a longer, more flexible clinical decision window, facilitating not only admission screening but also real-time risk assessment within the first 24 h.

In addition, sepsis is a highly heterogeneous disease characterized by complex “inflammation-coagulation” interactions^40^. Its progression involves multiple systems. Clinical classification based on multidimensional monitoring indicators and laboratory results has been a research hotspot recently. The predictive power of physiological and laboratory indicators alone early post-admission is limited for disease progression. The static nomogram established in this study achieved reliable baseline risk assessment early post-admission, but single-cross-section data may fail to fully capture the dynamic trajectory of disease progression. As highlighted in recent research^41^, the temporal characteristics of biomarkers (e.g., trajectory of progression) are crucial for accurately identifying clinical subtypes.

By placing the core indicators identified in this study on a time axis, we found that their dynamic fluctuation patterns may predict different outcomes, which points out the direction of our subsequent study: To transform the static screening of this study into dynamic risk monitoring using advanced statistical methods such as cluster analysis and growth models. This will help clinicians transition from ‘one-time assessment’ to ‘full disease trajectory management’.

Finally, some predictors identified by this model (e.g., Use. of. CAs, RT) are clinically modifiable risk factors. We confirmed their predictive value by sensitivity analyses, but their causality with prognosis remains to be established by more advanced statistical methods. As suggested by recent research^42^, the target trial emulation (TTE) framework can effectively resolve biases in observational studies, thereby simulating RCTs by real-world data. In future studies, we plan to incorporate TTE or causal mediation analysis to deeply explore the clinical benefits of precision interventions for key indicators identified by this model. This will help upgrade current risk assessment tools into more instructive clinical decision support systems.

## Limitations

The limitations of this study are primarily attributed to its retrospective design and the inherent nature of the database. First, large-scale public databases were utilized, but all data originated from the ICU, restricting the model’s generalizability to emergency departments outside the ICU. Moreover, some confounders were not measured. Due to the limitation of retrospective database structure, we could not know the specific cause of cirrhosis (e.g., alcoholic, viral, or metabolic), whether it was in the end stage, or whether advanced interventions such as long-term immunosuppressive therapy or TIPS were made. Undeniably, these differences in baseline conditions may not only introduce bias in baseline clinical indicators at admission but also result in fundamentally distinct risk profiles among patients with different causes of cirrhosis when complicated by sepsis. Due to the absence of detailed treatment background and etiological stratification, the model’s predictive efficacy may be restricted in specific subgroups. Therefore, future studies should conduct subgroup analyses based on precise stratification of baseline conditions and treatment histories in a larger prospective cohort, thereby further optimizing and validating the model’s predictive value. Second, the study population was predominantly Caucasian patients, possibly affecting the model’s applicability to other races. Third, methodologically, selection bias inherent in retrospective studies cannot be entirely ruled out. Despite rigorous data characterization and multiple imputation, the potential impact of selection bias on model results remains present. Furthermore, due to the heterogeneity in variable acquisition in eICU-CRD, only 352 patients were included in the external validation set, which to some extent weakened the interpretability of the model in external validation. The small sample size and high heterogeneity of the external validation set resulted in a lower AUC value than the training set. This suggests potential fluctuations in the model’s generalizability across centers due to variations in healthcare systems (e.g., admission criteria and resource allocation in different centers). In the future, large-scale, prospective, multicenter studies covering diverse ethnic backgrounds and multiple clinical settings are required to further refine and validate this model. We acknowledge that compared with complex “black-box” models like Gradient Boosting Machine (GBM), the model established in this study may exhibit limitations in predictive accuracy. However, we believe that this model strikes a favorable balance between predictive performance and clinical utility, with comparable interpretability and simplicity to classic clinical scores, which contributes more to clinicians’ understanding and popularization.

## Conclusion

In conclusion, a nomogram model was established and validated using 11 easily accessible parameters for predicting the all-cause IHM in cirrhosis with sepsis, which can provide guidance for clinical treatment and ameliorate prognosis.

## Supplementary Information

Below is the link to the electronic supplementary material.

Supplementary Material 1

Supplementary Material 2

Supplementary Material 3

Supplementary Material 4

Supplementary Material 5

Supplementary Material 6

Supplementary Material 7

## Data Availability

MIMIC-IV 3.1 (https//physionet.org/content/mimiciv/3.1) and eICU-CRD (https//physionet.org/content/eicu-crd/2.0/).

## Abbreviations

ICU: Intensive care unit
SQL: Structured query language
SOFA: Sepsis-related organ failure assessment
SAPS-II: Simplified Acute Physiology Score II

## Declarations

### Data sources

MIMIC-IV 3.1 (https//physionet.org/content/mimiciv/3.1) and eICU-CRD (https//physionet.org/content/eicu-crd/2.0/).
