# Supplementary material for: Development and external validation of a predictive model for in-hospital mortality in patients with liver cirrhosis and sepsis
Source: Sci Rep. 2026 Apr 2;16:15885. doi: 10.1038/s41598-026-43991-x (PMC13194707; doi:10.1038/s41598-026-43991-x)
Supplement: Supplementary file 1 — Supplementary Material 1 [file 41598_2026_43991_MOESM1_ESM.docx]

Table S1 Lasso Regression Variable Selection

| Variable | Coefficient before Lasso regression | Coefficient after Lasso regression | Selected (Yes/No) |
| --- | --- | --- | --- |
| Age | 0.00227 | 0.00899 | Yes |
| AKI | 0.217 | 0 | No |
| Albumin | -0.147 | 0 | No |
| ALT | -0.00351 | -0.00216 | Yes |
| Anion. Gap | -0.00335 | 0 | No |
| AST | 0.0000226 | 0 | No |
| CHF | 0.176 | 0 | No |
| Creatinine | -0.0305 | 0 | No |
| DM | -0.229 | 0 | No |
| INR | 0.316 | 0.21 | Yes |
| Lac | 0.163 | 0.128 | Yes |
| MBP | -0.0227 | 0 | No |
| Mechanical. ventilation | -0.0827 | 0 | No |
| Platelets | -0.000237 | 0 | No |
| Potassium | 0.00949 | 0 | No |
| Race | 0.189 | 0 | No |
| RBC | 0.0678 | 0 | No |
| RDW | 0.0651 | 0.042 | Yes |
| RR | 0.0412 | 0.02 | Yes |
| RT | 0.626 | 0.34 | Yes |
| Sex | -0.154 | 0 | No |
| Sodium | 0.0000107 | 0 | No |
| Temperature | -0.189 | -0.094 | Yes |
| Total. Bilirubin | 0.0349 | 0.0203 | Yes |
| Use.of.CA | 0.735 | 0.603 | Yes |
| Use.of.GCs | -0.306 | -0.0519 | Yes |
| Use.of.HAS | 0.15 | 0 | No |
| WBC | 0.00346 | 0 | No |

Note: STATUS refers to whether the patient died in the hospital. AKI, acute kidney injury; CHF, congestive heart failure; DM, diabetes mellitus; Mechanical ventilation, mechanical ventilation; HAS, human albumin injection; Use of GCs, use of glucocorticoids; Use of CA, use of catecholamines; SAPS-II, simplified acute physiology score.

Table S2 Sensitivity analysis of model performance between imputed dataset and complete cases

| Group | N | AUC | | CI _ Lower | CI _ Upper |
| --- | --- | --- | --- | --- | --- |
| Imputed _ Train _ Set | 1701 | 0.7829 | 0.7613 | | 0.8044 |
| Raw _ Complete _ Cases | 1701 | 0.7767 | 0.7562 | | 0.7973 |

Table S3 Comparison of the predictive performance between the Nomogram and SAPS-II score across three cohorts

| Cohort | n | | Nomogram AUC (95% CI) | | SAPS-II AUC (95% CI) | DeLong _ P_Value | |
| --- | --- | --- | --- | --- | --- | --- | --- |
| Training | | 1701 | | 0.783 (0.761–0.804) | 0.723(0.698 - 0.746) | | < 0.001 |
|  | |  | |  |  | |  |
| Internal Validation | | 729 | | 0.763 (0.729-0.796) | 0.741(0.706 - 0.777) | | 0.287 |
|  | |  | |  |  | |  |
| External Validation | | 352 | | 0.745 (0.692-0.797) | 0.798 (0.750–0.847) | | 0.080 |

Table S4 Sub-group analysis: Head-to-head comparison of AUCs among three predictive models

| Cohort | Model | n | AUC (95% CI) | DeLong _P_Value |
| --- | --- | --- | --- | --- |
| External Validation | Nomogram | 114 | 0.798 (0.672–0.925) | - |
|  | SAPS-II | 114 | 0.737 (0.622–0.852) | 0.196 |
|  | APACHE - II | 114 | 0.728 (0.609–0.846) | 0.138 |

Table S5 Multivariable Logistic Regression Analysis of Predictors Selected by LASSO

| Variable | Beta | OR (95% CI) | P _ value |
| --- | --- | --- | --- |
| Age | 0.022 | 1.022 (1.013-1.032) | <0.01 |
| RT | 0.562 | 1.754 (1.359-2.266) | <0.01 |
| Use. of. GCs | -0.307 | 0.735 (0.581-0.93) | 0.01 |
| Use.of.CA | 0.814 | 2.257 (1.692-3.033) | <0.01 |
| Temperature | -0.193 | 0.824 (0.731-0.928) | 0.002 |
| RR | 0.043 | 1.043 (1.02-1.067) | <0.01 |
| Lac | 0.162 | 1.176 (1.127-1.229) | <0.01 |
| RDW | 0.067 | 1.069 (1.028-1.112) | 0.001 |
| INR | 0.318 | 1.374 (1.2-1.585) | <0.01 |
| ALT | -0.003 | 0.997 (0.996-0.998) | <0.01 |
| Total. Bilirubin | 0.033 | 1.033 (1.019-1.048) | <0.01 |
