# Supplementary figures and images for: Development and external validation of a predictive model for in-hospital mortality in patients with liver cirrhosis and sepsis
Source: Sci Rep. 2026 Apr 2;16:15885. doi: 10.1038/s41598-026-43991-x (PMC13194707; doi:10.1038/s41598-026-43991-x)

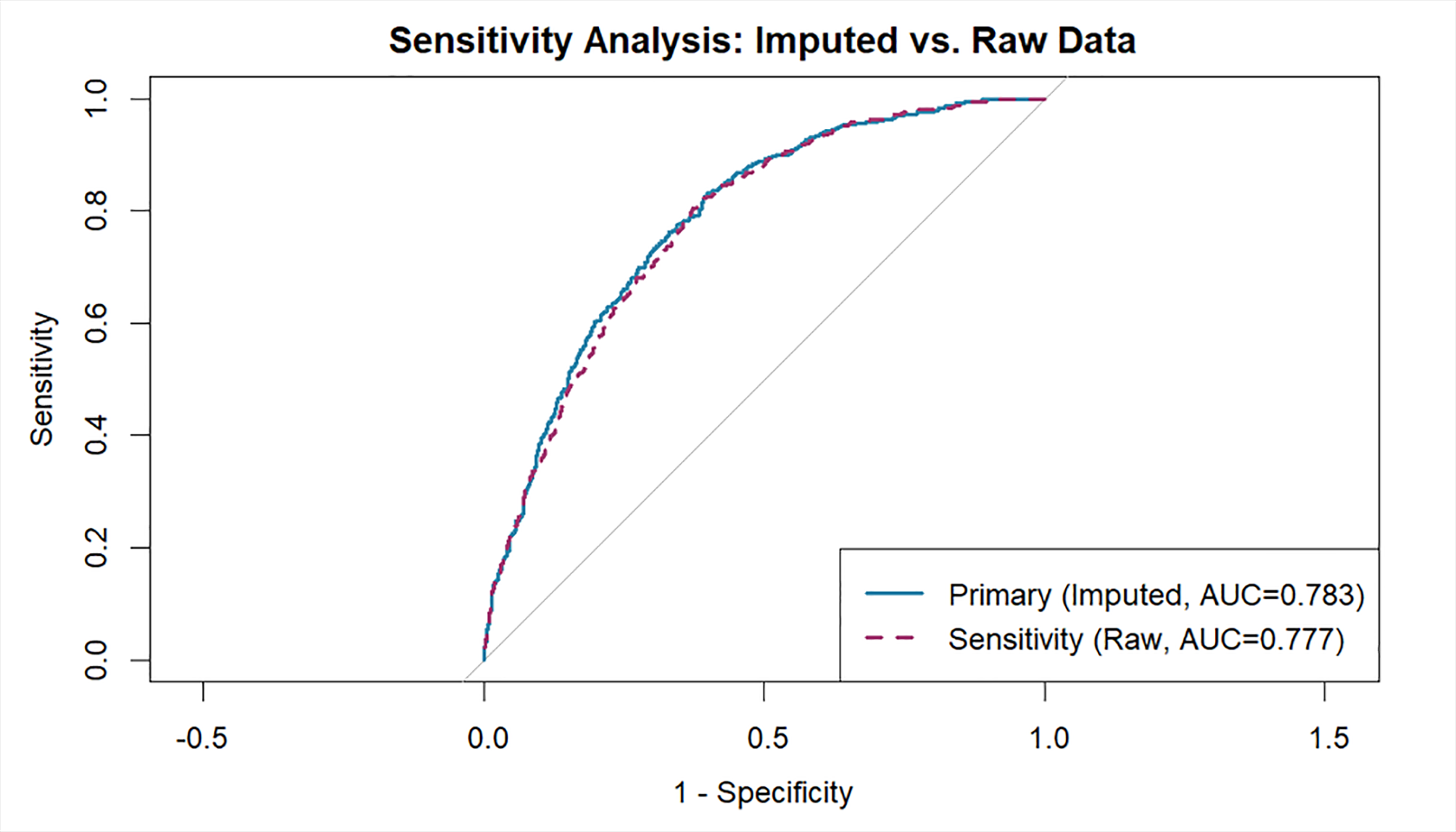

Supplement: Supplementary file 2 — Supplementary Material 2 [file 41598_2026_43991_MOESM2_ESM.tif]

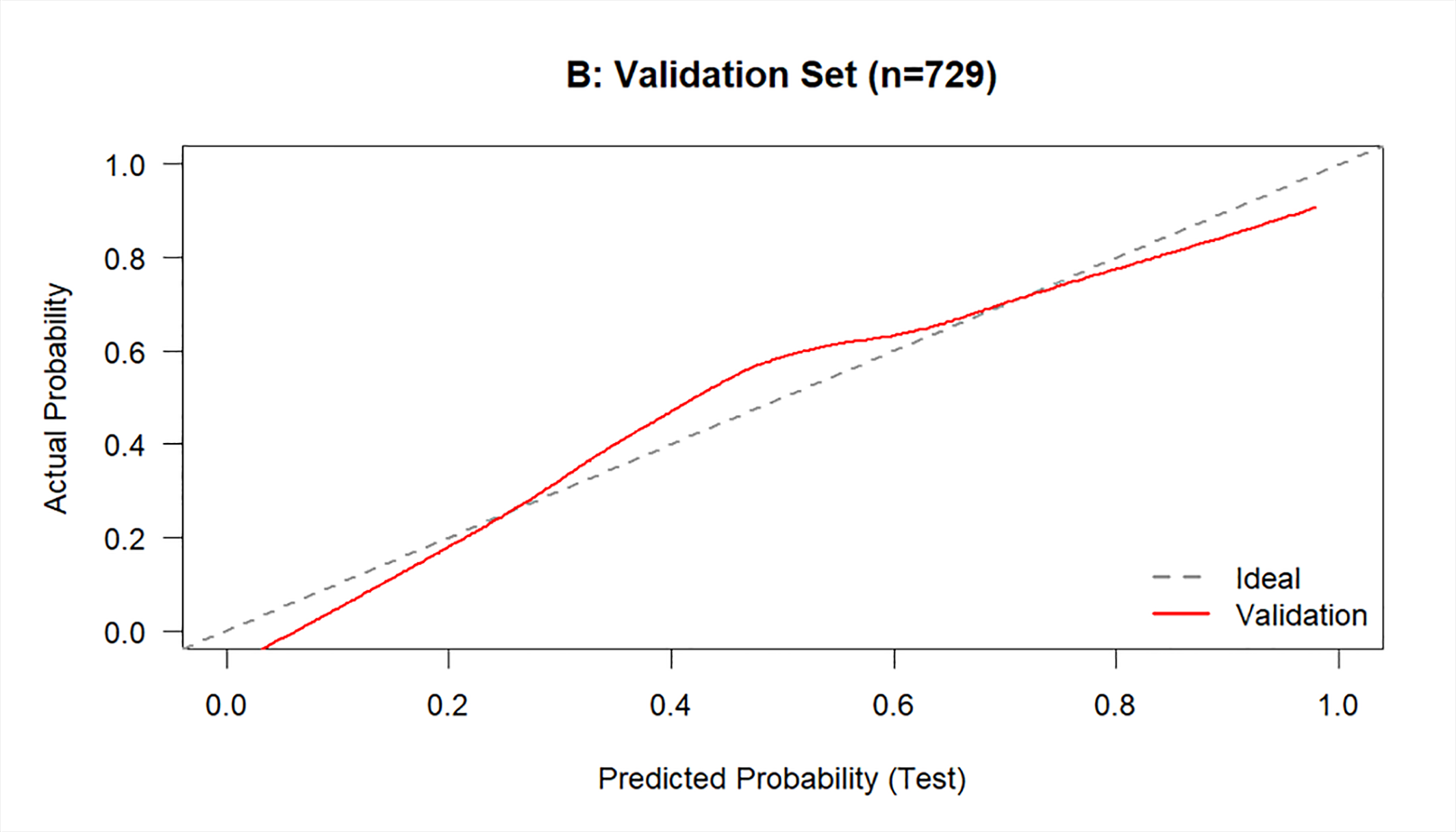

Supplement: Supplementary file 3 — Supplementary Material 3 [file 41598_2026_43991_MOESM3_ESM.tif]

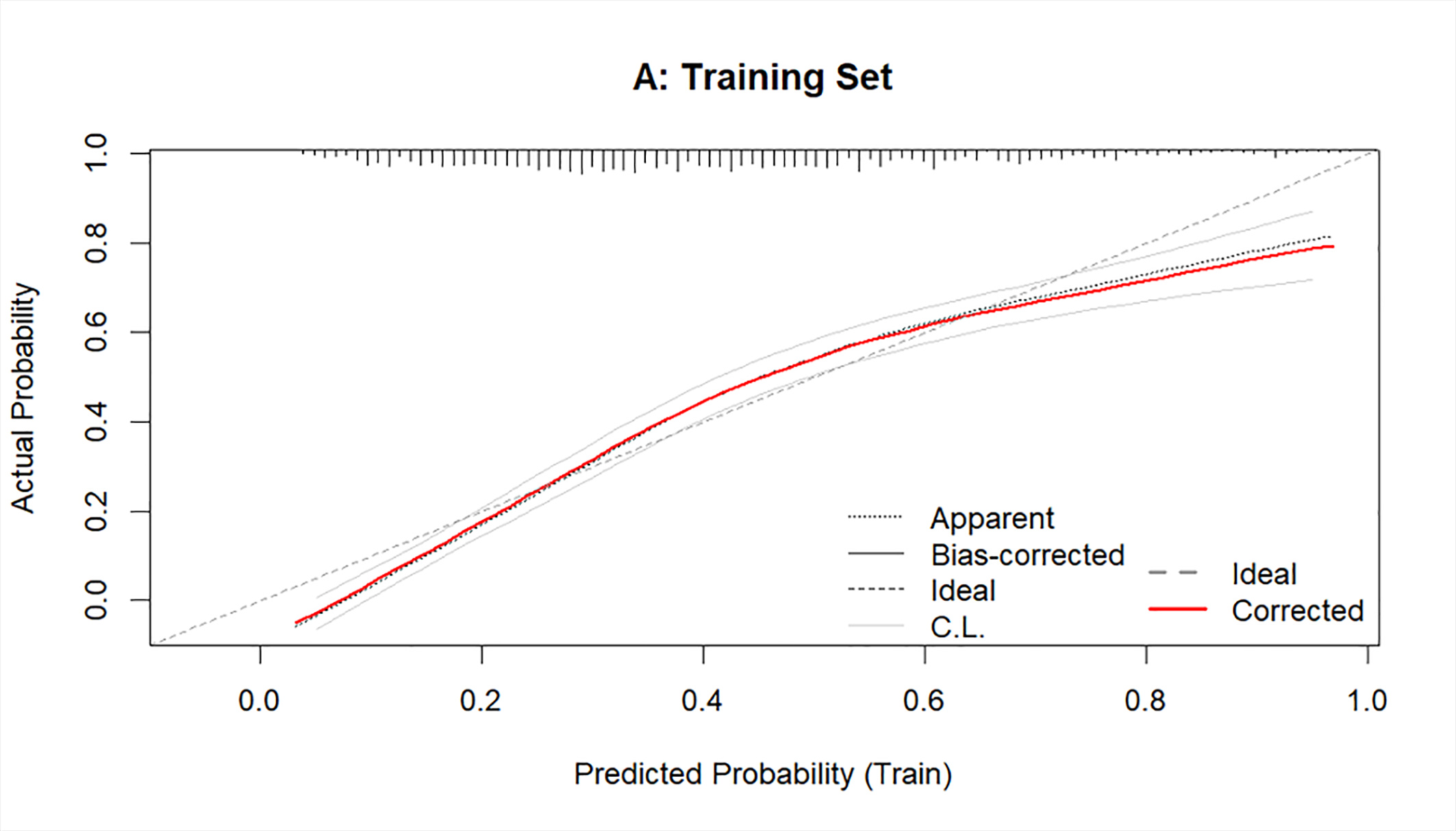

Supplement: Supplementary file 4 — Supplementary Material 4 [file 41598_2026_43991_MOESM4_ESM.tif]

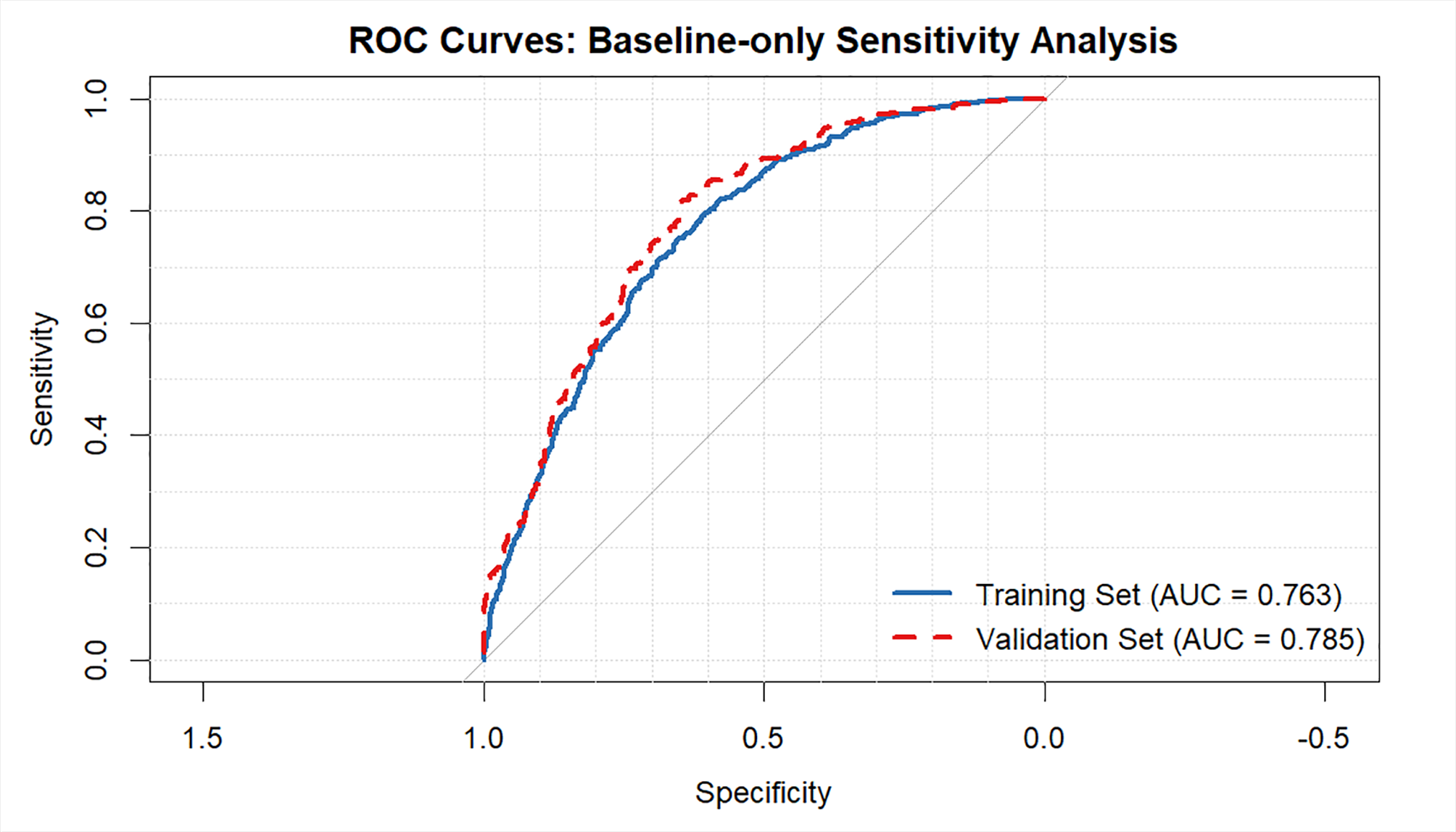

Supplement: Supplementary file 5 — Supplementary Material 5 [file 41598_2026_43991_MOESM5_ESM.tif]

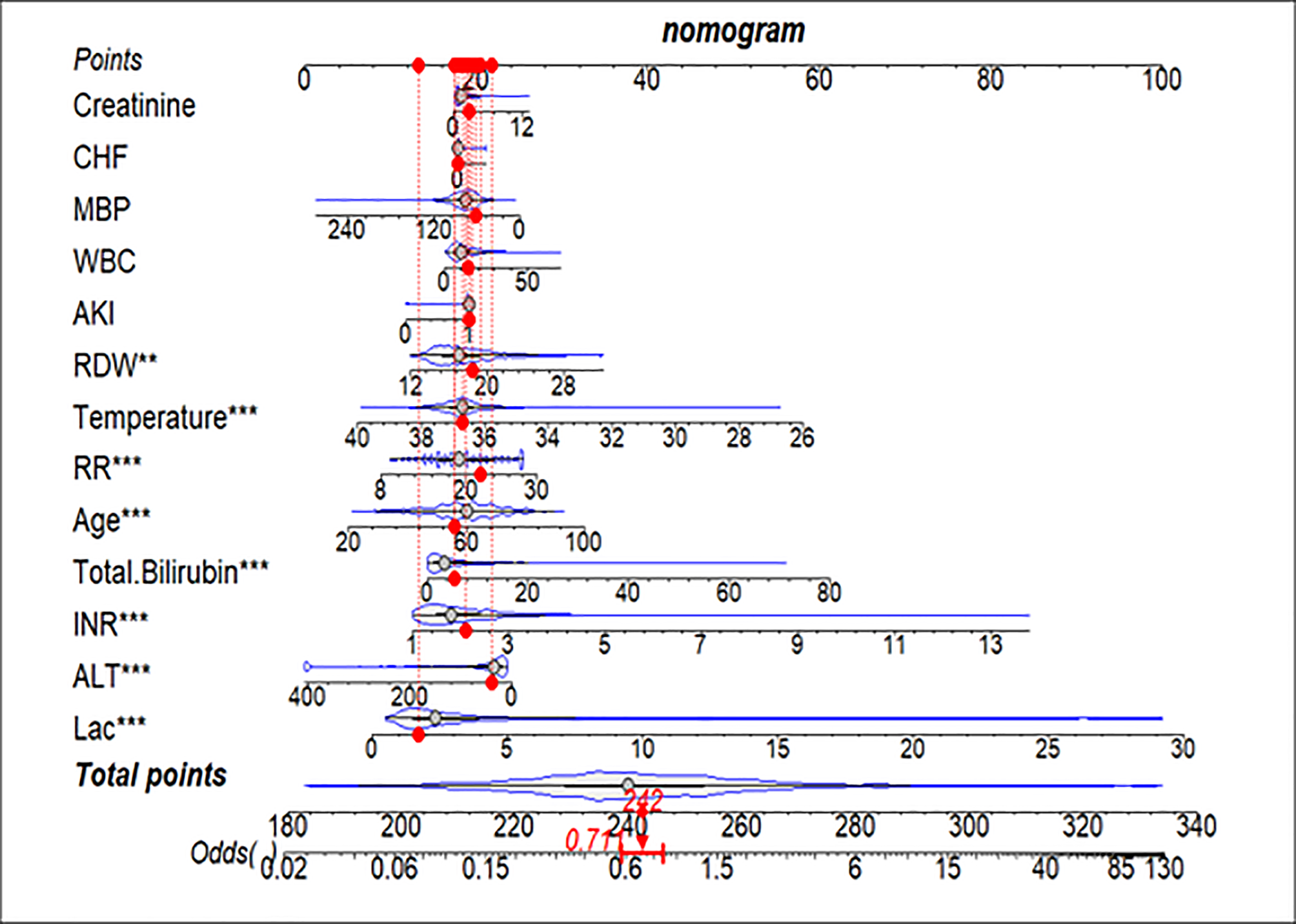

Supplement: Supplementary file 6 — Supplementary Material 6 [file 41598_2026_43991_MOESM6_ESM.tif]

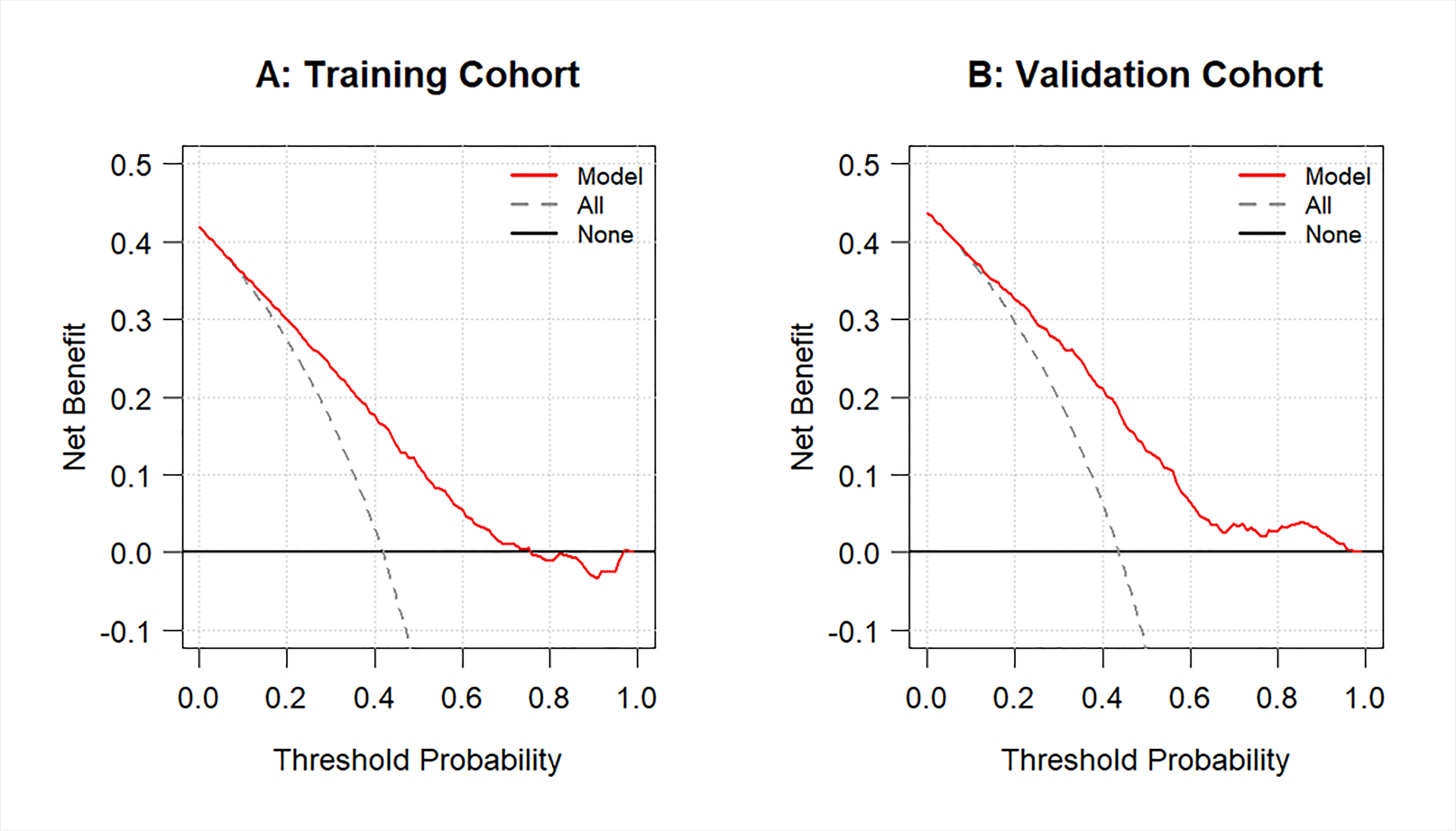

Supplement: Supplementary file 7 — Supplementary Material 7 [file 41598_2026_43991_MOESM7_ESM.tif]
